# Recent Advances in Preparing Transparent Phosphor Ceramics for High-Index Color Rendering and High-Power Lighting

**DOI:** 10.3390/molecules29061325

**Published:** 2024-03-16

**Authors:** Boshen Du, Wanyuan Li, Lin Zhang, Pei Chen, Fengniu Lu

**Affiliations:** 1 School of Materials Science and Engineering, Shaanxi Normal University, Xi’an 710119, China; 2 Department of Chemistry and Chemical Engineering, Beijing Institute of Technology, Beijing 100081, China

**Keywords:** transparent phosphor ceramics, high color rendering, high-power lighting, white LD, white LED

## Abstract

In recent years, high-power white light-emitting diode (wLED)/laser diode (wLD) lighting sources based on transparent phosphor ceramic (TPC) materials have attracted increasing application interest in automotive headlights, projection displays, and space navigation lighting due to their superior brightness, lighting distance, compactness, lifespan, and environmental resistance compared with the widely used phosphor-converted wLEDs. However, preparing TPC-converted wLEDs/wLDs with high color rendering index (CRI) remains a huge challenge, which limits their widespread application. In this review, we summarize the recently adopted strategies for constructing TPCs to develop high-power wLEDs/wLDs with high CRI values (>75). The construction protocols were categorized into four groups: host regulation, red-emitter doping, host regulation/red-emitter doping combination, and composite structure design. A comprehensive discussion was conducted on the design principles, photoluminescent properties, and device performances for each strategy. The challenges and future trends of high-power and high-CRI wLEDs/wLDs based on TPCs are also discussed toward the end of this review.

## 1. Introduction

The first decades of this century have witnessed an extraordinary revolution in the lighting industry, as blue light-emitting diodes (LEDs) were cleverly combined with yellow Y_3_Al_5_O_12_:Ce^3+^ (YAG:Ce^3+^) phosphors dispersed in organic resins to generate white light [1]. Due to their advantages of low energy consumption, non-pollution, and long service life, these phosphor-converted white LEDs (pc-wLEDs) have quickly replaced traditional incandescent and fluorescent lamps in a short time to become the mainstream lighting sources [2,3,4]. However, the thermal conductivity of organic resins is low, and thermal degradation occurs under the accumulated thermal radiation of the LED chip, which inevitably reduces the luminous efficiency (LE) and color quality of the pc-wLED devices during long-term use [5,6,7]. As the power of LEDs increases, this problem will worsen significantly. In order to achieve high-power lighting, alternative color converters with high thermal conductivity and strong resistance against thermal degradation are required.

Recently, a range of color converters for high-power wLEDs or white laser diode (wLD) lighting devices have been reported, including phosphor films [8,9,10,11,12], phosphors in glass [13,14,15,16,17,18,19,20,21,22], glass ceramics [23,24,25,26,27], single crystal phosphors [28,29,30,31,32,33], and transparent phosphor ceramics (TPCs) [34,35,36,37,38]. Among them, TPCs have gained particular attention due to their high thermal conductivity, excellent heat resistance, high mechanical strength, high homogeneity, and high LE [34,35,39,40,41]. The first TPC-converted wLED was reported in 2008 [42], in which YAG:Ce^3+^ ceramic was used. Since then, YAG:Ce^3+^ TPCs have been widely studied, and have shown broad application prospects in automotive headlights, projection displays, space navigation lighting, and other fields. However, the wLEDs/wLDs constructed with YAG:Ce^3+^ TPCs show low color rendering index (CRI) due to the lack of red emission component [34,38,40,43], which restricts their practical application.

To address the issue, many approaches to enhance the CRI values of YAG:Ce^3+^ TPC-converted wLEDs/wLDs have been explored in recent years. However, a systematic summary of this topic is still lacking. With the growing research interest in high-power and high-CRI lighting [16,44,45,46], a summary of the latest research results in this aspect is meaningful for junior researchers who are going to start their research in this area. Therefore, this review comprehensively summarizes the reported preparation methods for TPCs for constructing wLED/wLD lighting sources with high power and high CRI values (>75). The construction strategies were divided into four general categories: host regulation, red-emitter doping, host regulation/red-emitter doping combination, and composite structure design. For each strategy, design principles, photoluminescent (PL) properties, PL tuning mechanisms, and device performances were extensively discussed. Finally, the current challenges and future prospects of high-power and high-CRI wLED/wLD lighting systems are also presented. We believe that a comprehensive summary of construction strategies from foundation to application will benefit junior researchers in developing advanced lighting systems.

## 2. Modifying the YAG Host to Redshift the Emission or/and Widen the Emission Band of Ce^3+^

**Figure 1:**
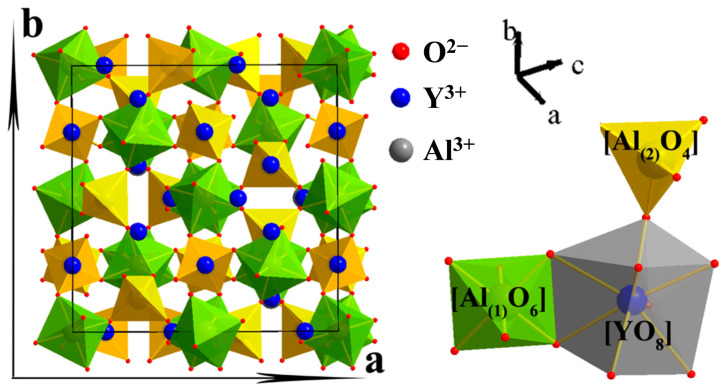
The crystal structure (**left**) of Y_3_Al_5_O_12_ and the coordination environment of Y^3+^ and Al^3+^ cations (**right**) in the lattice. Reprinted with permission from ref. [47]. Copyright 2014, American Chemical Society.

As a well-known phosphor host, YAG has a garnet structure (Figure 1) with a general formula of Y_3_Al(1)_2_Al(2)_3_O_12_, where Y, Al(1), and Al(2) are coordinated with 8, 6, and 4 oxygen atoms to form a dodecahedron, octahedron, and tetrahedron, respectively. The most widely used yellow phosphor YAG:Ce^3+^ is obtained by replacing a proportion of the Y^3+^ ions with Ce^3+^. Through further substitution/co-substitution of proper cations on the Y, Al(1), or Al(2) sites, the crystal field of the host lattice can be tailored, which results in PL tuning of YAG:Ce^3+^. Notably, the cation substitution occurs only when the coordination numbers (CNs) are equal and the ionic radii are similar. In order to better understand all material systems in this review, the ionic radii for a given CN of all relevant ions are listed in Table 1.

For YAG:Ce^3+^-based photo-converters, an effective way to add red emission components to their PL spectra is to adjust the crystal field of the 5d orbital of Ce^3+^, which is capable of shifting the emission peak of Ce^3+^ to longer wavelengths or/and broadening the full width at half maximum (FWHM) of the emission band. This can be accomplished by modifying the YAG garnet host via two general technical approaches: one is to replace the dodecahedral Y^3+^ site with ions with larger ionic radii, such as Gd^3+^ or Tb^3+^; the other is to replace octahedral/tetrahedral Al^3+^ with Ca^2+^-Si^4+^, Mg^2+^-Si^4+^, or Mg^2+^-Ge^4+^ ion pairs with longer bond lengths [48,49,50,51,52,53]. Using this strategy, a series of YAG:Ce^3+^-based ceramics with high CRI have been reported.

**Figure 2:**
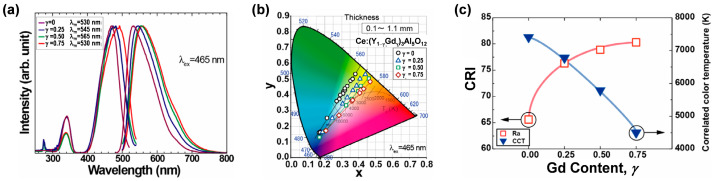
(**a**) PL and PL excitation (PLE) spectra of the (Gd*_γ_*Y_1−*γ*_)_3_Al_5_O_12_:Ce^3+^ (*γ* = 0–0.75) ceramics. (**b**) Chromaticity color coordinates of the samples under 465 nm LED excitation. (**c**) Gd content dependence of CRI and correlated color temperature (CCT) of the Ce:GdYAG ceramics. Reprinted with permission from ref. [54]. Copyright 2011, IOP Publishing.

In 2011, Nishiura et al. [54] synthesized a series of transparent (Gd*_γ_*Y_1-*γ*_)_3_Al_5_O_12_:Ce^3+^ (*γ* = 0–0.75) ceramics with thicknesses of 0.1–1.1 mm through the vacuum sintering technique. The 5d_1_ → 4f transition of Ce^3+^ led to broad PL emission bands for all samples. As the content of Gd^3+^ increased, the peak of the emission bands shifted from 530 nm to 560 nm (Figure 2a), and the sample color changed from greenish yellow to yellow, which can be attributed to the increase in crystal field splitting (CFS) upon the substitution of Y^3+^ with Gd^3+^. Under the excitation of a 465 nm LED, the color coordinates shifted from blue to the yellow or orange region upon increasing the thickness of the samples from 0.1–1.1 mm (Figure 2b). For samples with same thickness, the color coordinates redshifted with the increase of Gd^3+^ content. When connecting the color coordinates of the sample with the same Gd^3+^ content with a straight line, it was found that the straight lines of (Gd*_γ_*Y_1−*γ*_)_3_Al_5_O_12_:Ce^3+^ (*γ* = 0.25–0.75) passed through the theoretical white point of (0.33, 0.33), while that of the YAG:Ce^3+^ ceramic did not. In other words, by the substitution of Gd^3+^ for Y^3+^, the emission color can be adjusted to pure or warmer white upon optimization of the sample thickness. As summarized in Figure 2c, with the increase of the Gd^3+^ content, the CRI value of the samples increased from 65 to 81, indicating their suitability as promising candidate color converters for high-power wLED/wLD lighting.

To further investigate the LE of the material, in 2015, Chen et al. [55] also introduced Gd^3+^ into YAG:Ce^3+^ ceramics. The extent of redshift of the PL peak was proportional to the doping concentration of Gd^3+^. When combined with the InGaN blue chip, a CRI of up to 78.1 and a LE of 128.15 lm/W were obtained at an optimal Gd^3+^ concentration of around 10%.

**Figure 3:**
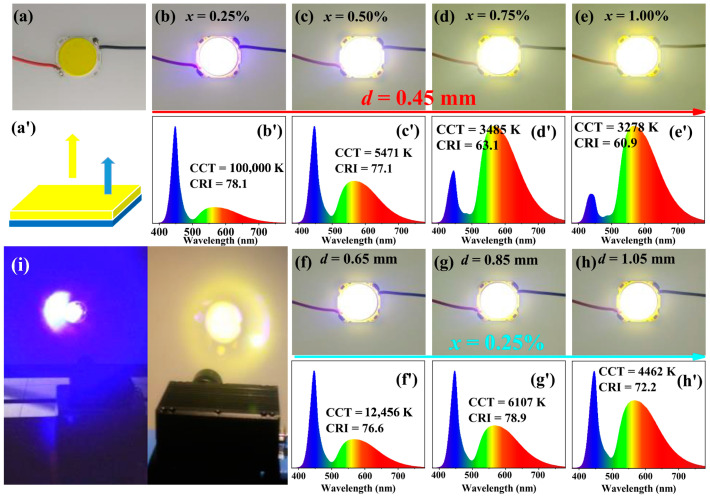
(**a**–**h**,**a’**) Photographs of GAGG:*x*Ce^3+^ ceramics on InGaN-based blue chips. (**b’**–**h’**) Electroluminescent spectra, CCT, and CRI of the obtained WLEDs. (**i**) Images of a 445 nm blue LD with 2 W (**left**) and a prototype wLD combining the blue LD and the GAGG:0.75%Ce^3+^ ceramic (thickness(*d*) = 1.05 mm) in operation (**right**). Reprinted with permission from ref. [56]. Copyright 2019, American Chemical Society.

Taking advantage of the rich red emission components and readily adjusted luminescent properties of (Gd,Y,Lu)_3_(Al,Ga)_5_O_12_:Ce^3+^ garnets [56], Liu et al. [57] designed and synthesized a series of highly transparent Gd_3_Al_4_GaO_12_:*x*Ce^3+^ (GAGG:*x*Ce^3+^, *x* = 0.25–1.00%) ceramics with thicknesses of 0.45, 0.65, 0.85, and 1.05 mm by sintering under an oxygen atmosphere. The broad emission bands (FWHM = 133–137 nm) and abundant red emission components (*λ*_em_ = 568–574 nm) of the GAGG:*x*Ce^3+^ ceramics enable warm white light upon excitation by blue LED or LD chips (Figure 3a–h,a’). According to Figure 3b’–h’, with the increase of the concentration of Ce^3+^ or thickness of the samples, more and more blue light emitted from the chips was absorbed, resulting in an increase in the emission ratio of GAGG:Ce^3+^. As a result, the white emission color shifted from cool to warm and the CRI reached 78.9. When fabricating GAGG:0.75%Ce^3+^ ceramic (thickness = 1.05 mm) with a 445 nm blue LD of 2 W, the strong blue light (Figure 3i, left) changed to warm white with a CCT of 3053 K (Figure 3i, right), indicating the feasibility of the material for high-power wLEDs/wLDs with a comfortable warm white light.

**Figure 4:**
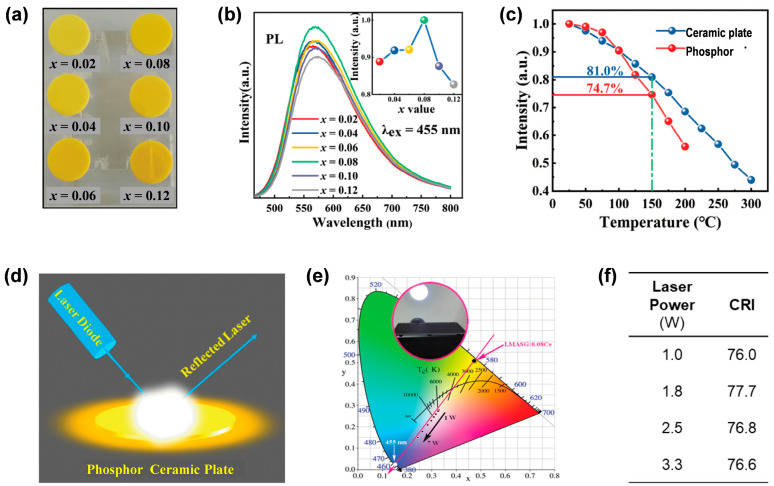
(**a**) Image of LMAS:*x*Ce^3+^ ceramic plates under daylight. (**b**) PL spectra (λ_ex_ = 455 nm) of LMAS:*x*Ce^3+^ ceramic plates. The inset shows the variation of PL peak intensities with different *x* values. (**c**) Temperature-dependent PL peak intensities of the ceramic plate and the phosphor of LMAS:0.08Ce^3+^. (**d**) Sketch map of wLD lighting with a reflection mode. (**e**) Chromaticity coordinates of the LMAS:0.08Ce^3+^ ceramic plate excited by different laser powers. Inset, a wLD device that has a maximum CRI of 77.7. (**f**) CRI of the LMAS:0.08Ce^3+^ ceramic plate driven by different laser powers. Reprinted with permission from ref. [58]. Copyright 2020, The Royal Society of Chemistry.

In 2020, Sun et al. [58] selected Lu_2_Mg_2_Al_2_Si_2_O_12_:Ce^3+^ (LMAS:Ce^3+^) phosphor with a broad emission band (λ_em_ = 575 nm, FWHM = 144 nm) [59] and synthesized LMAS:Ce^3+^ phosphor ceramic using the vacuum sintering technique. As shown in Figure 4a, a series of LMAS:*x*Ce^3+^ (*x* = 0.02–0.12) ceramic plates (diameter of 11 mm, thickness of 1 mm) was prepared. The samples showed broad emission bands spanning 475–800 nm when excited at 455 nm (Figure 4b), which could be ascribed to the 5d^1^ → 4f transition of Ce^3+^ ions (occupying the dodecahedral Lu^3+^ sites). When *x* = 0.08, the emission intensity reached the maximum value, displaying a broad yellow-orange peak at 565 nm with a FWHM of 130 nm. According to the thermal quenching properties shown in Figure 4c, the emission intensity of the LMAS:0.08Ce^3+^ ceramic plate at 150 °C decreased to 81.0% of the intensity at room temperature, while the pure LMAS:0.08Ce^3+^ phosphor decreased to 74.7% [59], indicating superior thermal stability of the ceramic plate compared with the phosphor. wLD light was obtained when LMAS:Ce^3+^ ceramic plates were fabricated with a blue LD in reflection mode (Figure 4d). When the laser power increased from 1 W to 3.3 W, the LMAS:0.08Ce^3+^ ceramic plate exhibited high CRI values (76.0–77.7) in consequence of the broadband emissive characteristic (Figure 4e,f).

## 3. Co-Doping Red-Emitting Cr^3+^, Pr^3+^, or Mn^2+^ Ions into YAG:Ce^3+^

Another approach to supplement the red-emission component of YAG:Ce^3+^ ceramics is to co-doping red-light-emitting ions such as Cr^3+^, Pr^3+^, or Sm^3+^ into the phosphor ceramic material.

### 3.1. Co-Doping Cr^3+^ Ions into YAG:Ce^3+^

In 2021, Lu et al. [60] prepared Ce^3+^ and Cr^3+^ co-doped Al_2_O_3_-YAG eutectics through the micro-pulling-down method. X-ray diffraction (XRD) and scanning electron microscopy and energy dispersive spectroscopy (SEM-EDS) results showed substitution of Y^3+^ with Ce^3+^ and occupation of octahedral Al^3+^ sites by Cr^3+^. The co-luminescence of Ce^3+^ (λ_em_ = 543 nm, 5d_1_ → 4f transition) and Cr^3+^ (λ_em_ = 694 nm, ^2^E → ^4^A_2_ transition) ions in Al_2_O_3_-YAG:Ce^3+^,Cr^3+^ significantly enhanced the red-emission component. However, since the emission band of Ce^3+^ overlapped with the absorption band of Cr^3+^, energy transfer occurred from Ce^3+^ to Cr^3+^, which quenched the yellow emission of Ce^3+^. TPC converted LED based on Al_2_O_3_-YAG:Ce^3+^,Cr^3+^ emitted only blue light from the LED chip and red light from Cr^3+^ ions. To address the issue, an Al_2_O_3_-YAG:Ce^3+^ and Al_2_O_3_-YAG:Cr^3+^ stacked TPC converted WLED was fabricated, the CRI of which reached 78.7.

**Figure 5:**
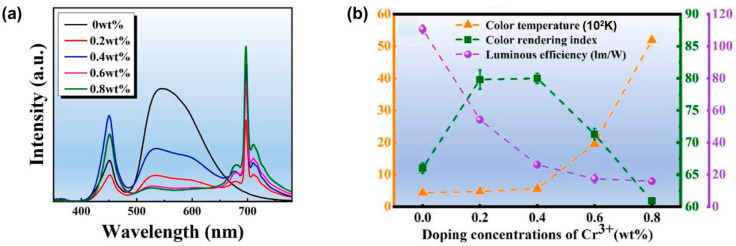
(**a**) The electroluminescent spectra of Ce/Cr co-doped Al_2_O_3_/YAG eutectic ceramics with different doping concentrations of Cr^3+^. (**b**) The CRI, CCT, and LE data of ceramics under a 175 mA driving current. Reprinted with permission from ref. [61]. Copyright 2022, Elsevier.

In 2022, Liu et al. [61] further investigated the influence of Cr^3+^ doping concentration on the luminescent properties of Ce/Cr co-doped Al_2_O_3_/YAG eutectic phosphor ceramics. The ceramics were prepared and fabricated through high-temperature sintering and the laser floating zone melting method. Upon adjustment of the concentrations of Cr^3+^ (0.2–0.8 wt%), the Ce^3+^ → Cr^3+^ energy transfer and luminescent properties of the ceramics were finely tuned. As shown in Figure 5a, after Cr^3+^ doping, strong red light emission centered at 694 nm appeared, which increased with the increase in Cr^3+^ concentration. At the same time, the emission intensity of Ce^3+^ around 550 nm decreased dramatically due to enhanced energy transfer from Ce^3+^ to Cr^3+^. As the concentration of Cr^3+^ increased, CRI first increased and then gradually decreased (Figure 5b), which can be rationalized by the suppression of yellow light emission. When the concentration of Cr^3+^ was equal to 0.4 wt%, CRI reached a maximum value of 80. Despite the improvement in CRI, overall LE declined (Figure 5b).

**Figure 6:**
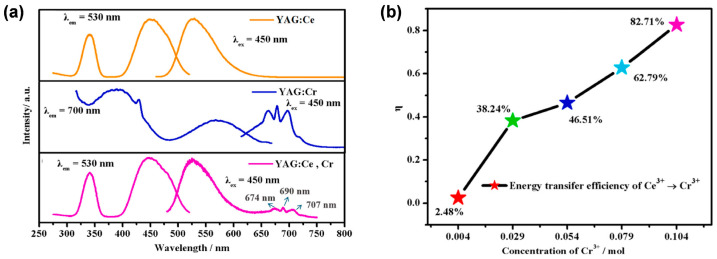
(**a**) PLE and PL spectra of YAG:Ce^3+^ (yellow), YAG:Cr^3+^ (blue), and YAG:Ce^3+^,Cr^3+^ (magenta). (**b**) CRI change trend graph of Y_2.94_Al_5−*x*_Cr*_x_*O_12_:0.06Ce^3+^ (*x* = 0.004, 0.029, 0.054, 0.079, 0.104 mol). Reprinted with permission from ref. [62]. Copyright 2023, Elsevier.

In 2023, Li et al. [62] prepared Y_2.94_Al_5−*x*_O_12_:0.06Ce^3+^,*x*Cr^3+^ (*x* = 0.004, 0.029, 0.054, 0.079, 0.104 mol) ceramics, in view of the wide emission band of Cr^3+^ spanning from 650 to 750 nm. As shown in Figure 6a, weak emission bands from 675 to 720 nm appeared upon the doping of Cr^3+^ into YAG:0.06Ce^3+^ ceramics. With the increase in the Cr^3+^ content, the CRI displayed a remarkable increase from 65.1 to 79.4 (Figure 6b). Therefore, the Ce^3+^, Cr^3+^ co-doped garnet ceramics are promising candidates for high-CRI lighting applications of wLEDs/wLDs.

### 3.2. Co-Doping Mn^2+^ Ions into YAG:Ce^3+^

**Figure 7:**
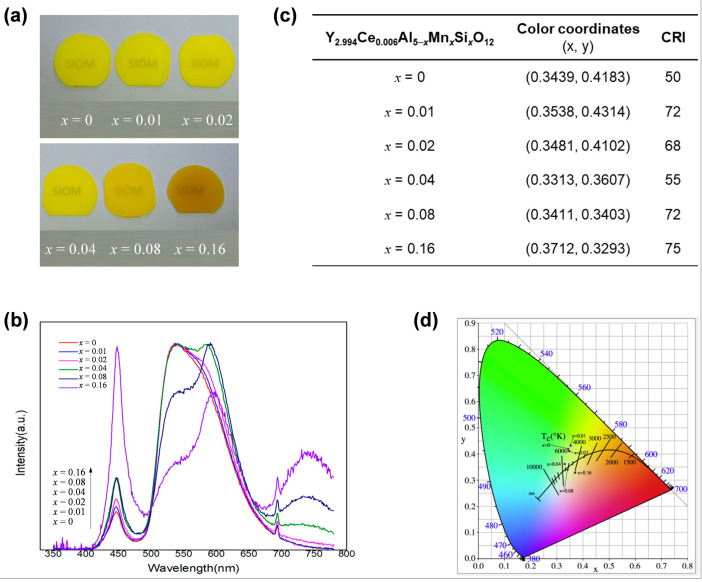
(**a**) Photograph of the YAG:Ce^3+^,*x*Mn^2+^,*x*Si^4+^ (*x* = 0, 0.01, 0.02, 0.04, 0.08, 0.16) phosphor ceramics. (**b**) Electroluminescent spectra of YAG:Ce^3+^,*x*Mn^2+^,*x*Si^4+^ coupled with 450 nm GaN blue chips. (**c**) The color coordinates and CRI of the packaged LEDs. (**d**) The CIE chromaticity diagram of YAG:Ce^3+^,*x*Mn^2+^,*x*Si^4+^ samples. Reprinted with permission from ref. [63]. Copyright 2019, Elsevier.

As summarized in Table 1, Mn^2+^ possesses multiple coordination numbers (CN) and feasibly occupies the tetrahedral Al^3+^ site, octahedral Al^3+^ site, and the dodecahedral Y^3+^ site in YAG host. Due to the spin-forbidden ^4^T_1_ → ^6^A_1_ d-d transition of Mn^2+^ into different occupation sites in the YAG lattice, the emission of Mn^2+^ can span from green to deep red depending on the specific coordination environment. In particular, Mn^2+^ at the octahedral position emits orange-red light, which is beneficial for promoting CRI. In this regard, Ao et al. [63] introduced Mn^2+^ into YAG:Ce^3+^ ceramics by preparing a series of Y_2.994_Ce_0.006_Al_5−2*x*_Mn*_x_*Si*_x_*O_12_ (YAG:Ce^3+^,*x*Mn^2+^,*x*Si^4+^) (*x* = 0, 0.01, 0.02, 0.04, 0.08, 0.16) ceramics via solid-state reaction under vacuum conditions (Figure 7a). The co-doping of Si^4+^ was for charge compensation. XRD data, together with the PL properties of Y_3_Al_4.98_Mn_0.02_O_12_, indicated that Mn^2+^ ions tended to occupy the position of octahedral Al^3+^, while Si^4+^ ions were located at the position of tetrahedral Al^3+^. As shown in Figure 7b, upon the doping of Mn^2+^/Si^4+^ pairs, the green emission band of Ce^3+^ (535 nm) as well as orange (587 nm, Mn^2+^ occupying the octahedron Al^3+^ site) and red (740 nm, Mn^2+^ occupying the dodecahedron Y^3+^ site) emission bands were observed, indicating the successful complementing of the red emission. It is worth noting that the emission band centered at 740 nm exceeds the cutoff wavelength (690 nm) of CIE 1931 color matching function, as the human eye cannot effectively perceive anything beyond 690 nm. Therefore, in wLEDs/wLDs, only the orange-red emission at 587 nm has significance in improving the CRI. As the concentration of Mn^2+^/Si^4+^ pairs increased, the emission intensity of Ce^3+^ decreased, while the emission intensity ratio of Mn^2+^ to Ce^3+^ increased (Figure 7b). The results indicate efficient energy transfer from Ce^3+^ to Mn^2+^, which is further evidenced by the shortened lifetime of Ce^3+^ in fluorescence lifetime decay studies. This energy transfer facilitates the realization of tunable emission color from yellow toward the orange-red region, as demonstrated by the changes of CIE chromaticity coordinates (x, y) in Figure 7c,d. When *x* = 0.16, a CRI as high as 75 was obtained, which is promising for warm white light application.

### 3.3. Co-Doping Cr^3+^ and Pr^3+^ Ions into YAG:Ce^3+^

**Figure 8:**
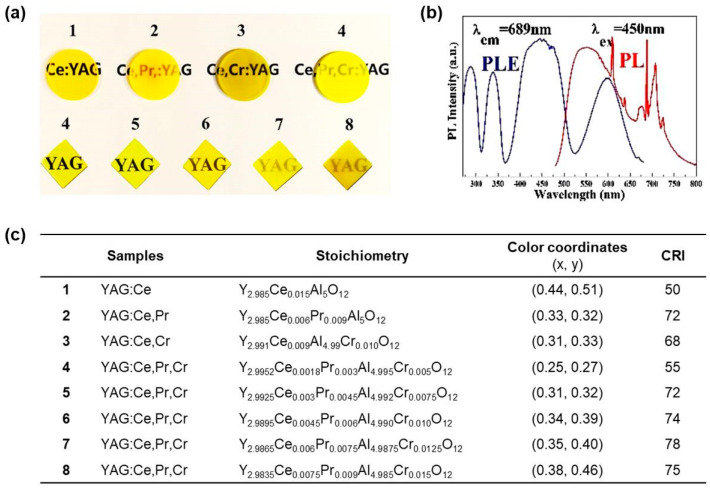
(**a**) Picture of as-prepared transparent ceramics **1**–**8**. (**b**) PL and PLE spectra of YAG:Ce^3+^,Pr^3+^,Cr^3+^. (**c**) Ingredients, color coordinates, and CRI of all the transparent ceramic packaged LEDs. Reprinted with permission from ref. [64]. Copyright 2017, Elsevier.

Pr^3+^ ion is a well-known orange-red light emitter upon irradiation of blue LEDs. Therefore, similar to the Ce^3+^,Cr^3+^ co-doped garnet ceramics, the doping of Pr^3+^ into YAG:Ce^3+^ ceramic can also supply the red spectral component and improve CRI performance. In 2017, Feng et al. [64] introduced both Cr^3+^ and Pr^3+^ into YAG:Ce^3+^ ceramics to expand the emission spectrum. The YAG:Ce^3+^,Pr^3+^,Cr^3+^ ceramics were prepared through a solid-state reaction–vacuum sintering approach. The introduction of Pr^3+^ and Cr^3+^ changed the yellow color of YAG:Ce^3+^ ceramic to orange-yellow and yellow-green (Figure 8a). Analysis of the XRD diffraction patterns confirmed that Ce^3+^ and Pr^3+^ occupied the position of Y^3+^, and Cr^3+^ resided on the position of octahedral Al^3+^. Under 450 nm excitation, characteristic emission peaks of Ce^3+^, Pr^3+^, and Cr^3+^ at 530, 609, and 689 nm appeared, enabling wide emission spectra spanning 500 to 750 nm (Figure 8b). Investigation of the fluorescence decay curves confirmed energy transfers of Ce^3+^ → Pr^3+^ and Ce^3+^ → Cr^3+^, consistent with the studies by Lu et al. [60]. Through systematic optimization of the doping concentration of Ce^3+^, Pr^3+^, and Cr^3+^, high-quality white light with CRI reaching 78 (Figure 8c) was obtained by fabricating the YAG:Ce^3+^,Pr^3+^,Cr^3+^ ceramics with blue LED.

### 3.4. Co-Doping Pr^3+^ and Mn^2+^ Ions into YAG:Ce^3+^

**Figure 9:**
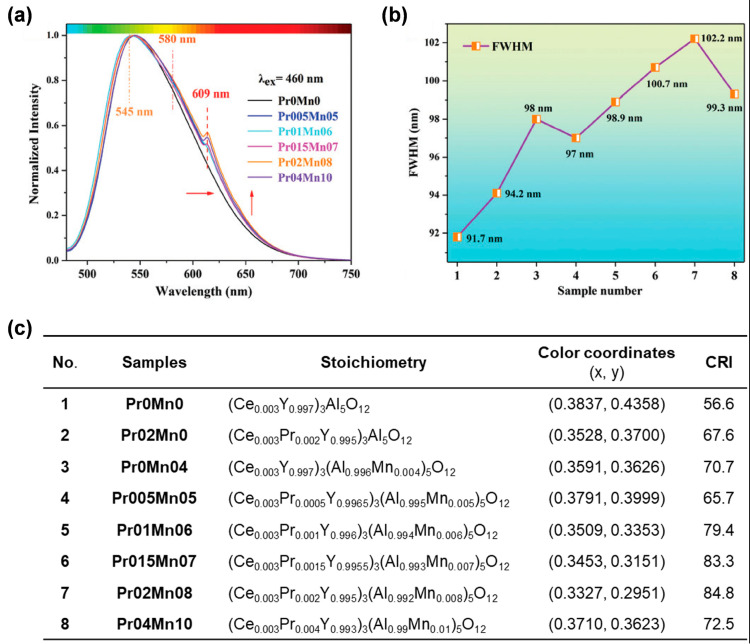
(**a**) Normalized PL spectra and (**b**) the FWHM evolution of the PL spectra of the prepared ceramics. (**c**) Ingredients, color coordinates, and CRI of all the ceramic packaged LEDs. Reprinted with permission from ref. [65]. Copyright 2020, The Royal Society of Chemistry.

In view of the orange light emission from Mn^2+^ and red light emission of Pr^3+^, Ma et al. [65] doped Mn^2+^ and Pr^3+^ synchronously into YAG:Ce^3+^ ceramics using a solid-state vacuum sintering technique. Due to the similarity of ion radii, in the YAG lattice, Pr^3+^ (1.126 Å, CN = 8) occupied the position of dodecahedral Y^3+^ (1.019 Å, CN = 8), while Mn^2+^ (0.67 Å, CN = 6) took up the position of octahedral Al^3+^ (0.535 Å, CN = 6), which was further confirmed by XRD patterns. The as-prepared single-structured YAG:Ce^3+^,Mn^2+^,Pr^3+^ ceramics exhibited typical emission peaks of Ce^3+^ (545 nm), Mn^2+^ (580 nm), and Pr^3+^ (609 nm) in the fluorescent spectra (Figure 9a). As the concentrations of Mn^2+^ and Pr^3+^ were adjusted, the FWHM of the emission spectrum increased from 91.7 nm to 102.2 nm (Figure 9b), which was conducive to achieving high CRI. When YAG:Ce^3+^,Mn^2+^,Pr^3+^ ceramics were assembled with a blue LED, the obtained CRI of the wLED device reached 84.8 for **Pr02Mn08** (Figure 9c).

## 4. Combining the Strategies of 2 and 3

As presented above, the red emission composition of YAG:Ce^3+^ ceramics can be increased by adjusting the YAG lattice to modulate the crystal field of the Ce^3+^ 5d orbit, or by adding red-light-emitting ions through synchronous doping. Attempts were also made to explore the synergistic effects of these two strategies.

### 4.1. Co-Doping Mn^2+^ Ions into TAG:Ce^3+^

**Figure 10:**
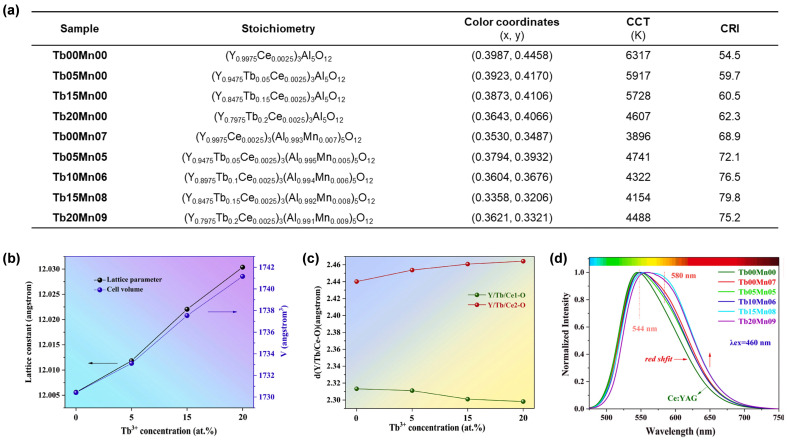
(**a**) Ingredients, color coordinates, CCT, and CRI of the YTAMG:Ce^3+^ ceramics. (**b**) The variation of lattice parameters and volume of **Tb00Mn00**–**Tb20Mn00** with Tb^3+^ concentration. (**c**) The Y/Tb/Ce1-O and Y/Tb/Ce2-O bond lengths of YTAMG:Ce^3+^ ceramics as a function of Tb^3+^ concentration. (**d**) Normalized PL spectra of all the YTAMG:Ce^3+^ ceramics (λ_ex_ = 460 nm). Reprinted with permission from ref. [52]. Copyright 2021, Elsevier.

In 2021, Ma et al. [52] prepared a series of (Y,Tb)_3_(Al,Mn)_5_O_12_:Ce (YTAMG:Ce^3+^) ceramics with different concentrations of Tb^3+^ and Mn^2+^ (Figure 10a) through a vacuum sintering technique. Due to the larger ionic radius of Tb^3+^ (1.04 Å, CN = 8) compared with that of Y^3+^ (1.019 Å, CN = 8), replacing Y^3+^ with Tb^3+^ will strengthen the crystal field around the Ce^3+^ ion. As confirmed by the refinement of the crystal structure of YTAMG:Ce^3+^ ceramics, the CFS of Ce^3+^ was enhanced via Tb^3+^ doping (Figure 10b,c). The promotion of CFS resulted in redshift of the emission band of Ce^3+^. In addition, the orange-red emission of Mn^2+^ (occupying the octahedral Al^3+^) further compensates for the red emission component. As a result of the dual effects, the emission band of the YTAMG:Ce^3+^ ceramics exhibited a redshift of 12 nm and an increase in FWHM (16.2%) compared with that of the YAG:Ce^3+^ ceramic (**Tb00Mn00**) (Figure 10d). When YTAMG:Ce ceramics were fabricated with blue LED chips, the resulting wLED exhibited a CRI of 79.8 for **Tb15Mn08**.

**Figure 11:**
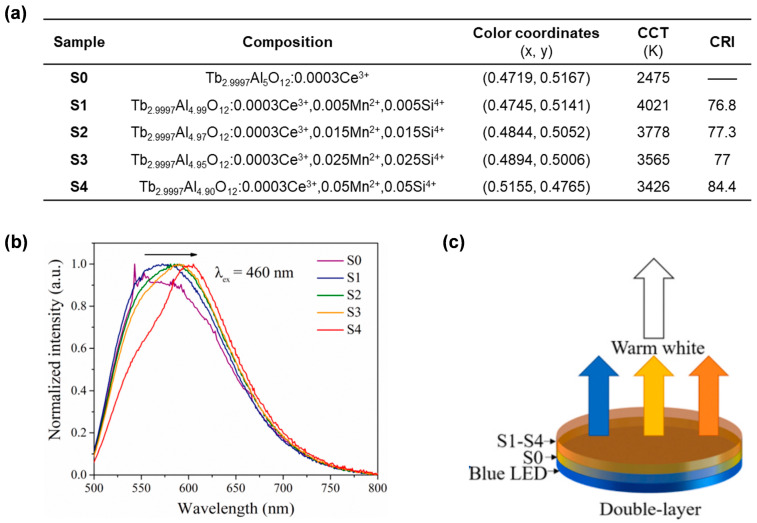
(**a**) Compositions, color coordinates, CCT, and CRI of the **S0**–**S4** ceramics. (**b**) Normalized PL spectra of **S0**–**S4** (λ_ex_ = 460 nm). (**c**) Schematic diagram of wLEDs based on the double-layer ceramic structure. Reprinted with permission from ref. [66]. Copyright 2021, Elsevier.

In another work presented by Duan et al. [66], Y^3+^ was not partially but instead completely replaced with Tb^3+^. They prepared a series of Tb_2.997_Al_5−2*x*_Si*_x_*O_12_:0.003Ce^3+^,*x*Mn^2+^ (**S0**–**S4**) phosphor ceramics with different contents of Mn^2+^-Si^4+^ (Figure 11a) via high-temperature solid-state reaction under oxygen. The synergistic effect of Tb^3+^ and Mn^2+^ resulted in orange-red emission in the case of **S4**, with an emission peak reaching 605 nm (Figure 11b). It is worth noting that wLED devices with high CRI (76.8–84.4) have been achieved by using a double-layer ceramic structure, where **S0** is installed on the blue LED chip and **S1**–**S4** are located on **S0** respectively (Figure 11c).

### 4.2. Co-Doping Mn^2+^ Ions into LuAG:Ce^3+^

Although modifying the YAG lattice with ions with larger ionic radii or ion pairs with longer bond lengths can redshift and/or widen the emission band of Ce^3+^, the thermal stability of the ceramic may be reduced if there is a large ion mismatch between the doped ions and the Y^3+^/Al^3+^ ions in the YAG lattice. In contrast, if the Y^3+^ ions in the YAG are completely replaced with Lu^3+^ ions with smaller ionic radius to form Lu_3_Al_5_O_12_ (LuAG), the thermal stability can be significantly improved [67]. In addition, Lu_3−*x*_Al_5_O_12_:*x*Ce^3+^ (LuAG:Ce^3+^) exhibits greater absorption coefficient and higher quantum yield than YAG:Ce^3+^ [68,69,70,71]. Therefore, LuAG:Ce^3+^ ceramics have excellent heat-quenching performance and broad application prospects in automotive headlights, biomedical equipment, and projection systems. However, as LuAG:Ce^3+^ ceramics emit green light, the lack of red emission components in the PL spectra severely limits their practical application.

**Figure 12:**
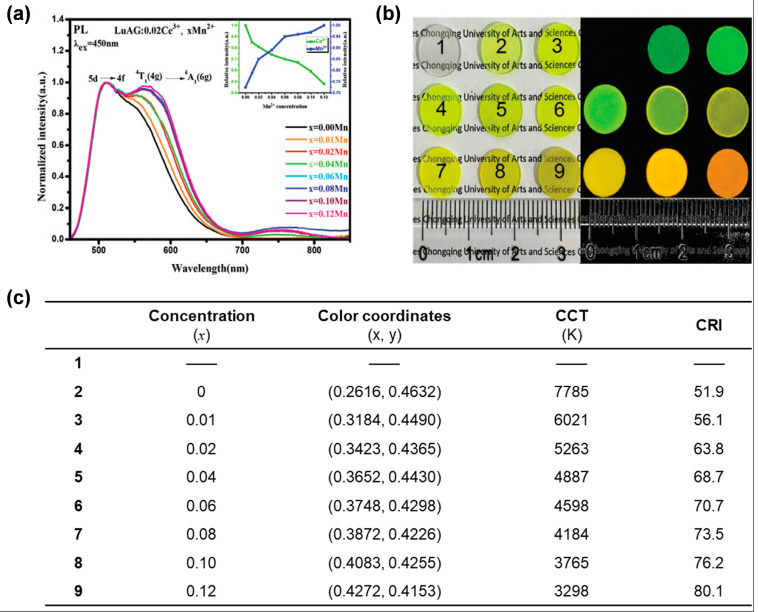
(**a**) PL spectra (λ_ex_ = 450 nm), (**b**) photos taken under natural light (**left**) and ultraviolet light (**right**), and (**c**) color coordinates, CCT, and CRI of the Lu_2.98_Al_5−2*x*_S*i_x_*O_12_:0.02Ce^3+^,*x*Mn^2+^ (*x* = 0–0.12) ceramics. Reprinted with permission from ref. [72]. Copyright 2020, The Royal Society of Chemistry.

Since hexacoordinate Mn^2+^ ions display orange-red emission (^4^T_1_ → ^6^A_1_ transition of Mn^2+^) [72,73] and are feasible to be doped into LuAG:Ce^3+^ ceramics [70,74], Yang et al. prepared a series of LuAG:Ce^3+^,Mn^2+^ ceramics through a solid-state vacuum sintering technique [75]. The occupation of the octahedral Al^3+^ sites by Mn^2+^ ions enables orange light emission peaking at around 588 nm due to the strong crystal field (Figure 12a). As the content of Mn^2+^ increases, a small number of Mn^2+^ ions occupy the dodecahedron Lu^3+^ sites, resulting in far-red emission light peaking at 750 nm. In addition, due to the efficient energy transfer from Ce^3+^ to Mn^2+^ (5d_1_ → ^4^T_1_), with the increase of Mn^2+^ concentration, the emission intensity of the Ce^3+^ ions (5d_1_ → 4f, occupying the dodecahedral Lu^3+^ sites) at 505 nm decreases, while the emission intensity of Mn^2+^ at 588 nm increases (Figure 12a, inset). As a result, by controlling the doping concentration of Ce^3+^ and Mn^2+^-Si^4+^ (Si^4+^ ions as charge balancer to replace tetrahedral Al^3+^ sites), the emission spectrum of Lu_2.98_Al_5−2*x*_Si*_x_*O_12_:0.02Ce^3+^,*x*Mn^2+^ (*x* = 0–0.12) can be adjusted in the range of 500 to 800 nm (Figure 12a), almost covering visible light. Under ultraviolet irradiation, the samples emit color from green to yellow and orange (Figure 12b). In addition, the materials exhibit excellent thermal stability. When *x* = 0.01, the PL properties remain at 99% of the initial value at 425 K. When assembled with high-power blue LDs, the sample (*x* = 0.12) has a high CRI of 80.1 (Figure 12c), a correlation CCT of 3298 K, and an LE of 68 lm/W, showing good application potential in museum lighting.

**Figure 13:**
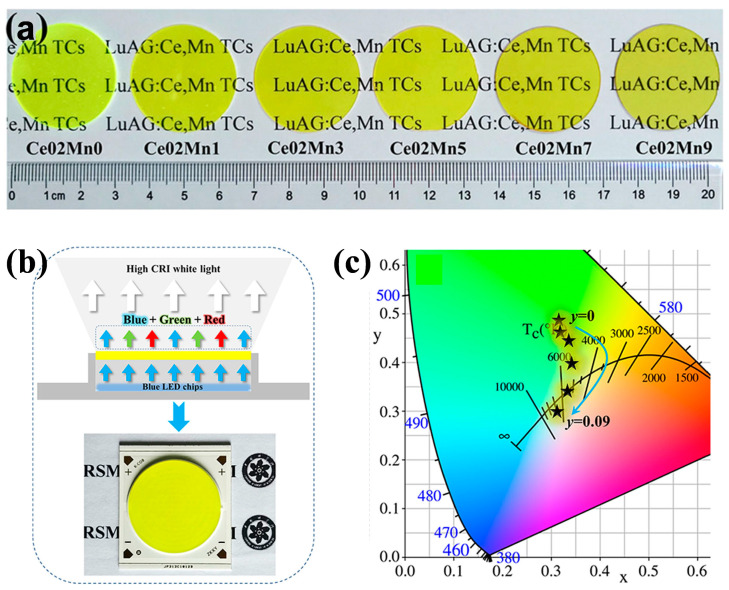
(**a**) Photograph of as-prepared LuAG:Ce,Mn phosphor ceramics. (**b**) Schematic diagram of high-CRI wLED encapsulation and actual chip-on-board lighting source. (**c**) The CIE chromaticity coordination diagram of these fabricated LEDs. Reprinted with permission from ref. [76]. Copyright 2022, The American Ceramic Society.

To further improve the LE and CRI of the above-mentioned LuAG:Ce^3+^,Mn^2+^ ceramics, Ling et al. [76] also co-doped Ce^3+^ and Mn^2+^ into LuAG-based ceramics. A series of Lu_2.998_Al_5−2*y*_Si*_y_*O_12_:0.002Ce^3+^,*y*Mn^2+^ (*y* = 0, 0.01, 0.03, 0.05, 0.07, 0.09) was synthesized via solid-state vacuum sintering, denoted as **Ce02Mn0**, **Ce02Mn1**, **Ce02Mn3**, **Ce02Mn5**, **Ce02Mn7**, and **Ce02Mn9**, respectively (Figure 13a). By carefully adjusting the occupied sites of Mn^2+^, the proportion of tricolor (orange, green, and blue) components can be changed, and the red emission centered at 590 nm and 750 nm can be effectively compensated. As a result, CRI achieved significant improvement. After assembling the LuAG:Ce^3+^,Mn^2+^ ceramics with blue LED, the variation of chromaticity parameters with Mn^2+^ doping concentration was systematically investigated (Figure 13b,c). When ceramic **Ce02Mn7** was applied, pure white light with a CRI of up to 91.0 and an LE of up to 85.07 lm/W was obtained. Unfortunately, the reason for the improvements in CRI and LE of Lu_2.998_Al_5−2*y*_Si*_y_*O_12_:0.002Ce^3+^,*y*Mn^2+^ (*y* = 0.07) compared with Lu_2.98_Al_5−2*x*_Si*_x_*O_12_:0.02Ce^3+^,*x*Mn^2+^ (*x* = 0.12) [72] was not elucidated.

### 4.3. Co-Doping Sm^3+^ and Mn^2+^ Ions into LuAG:Ce^3+^

**Figure 14:**
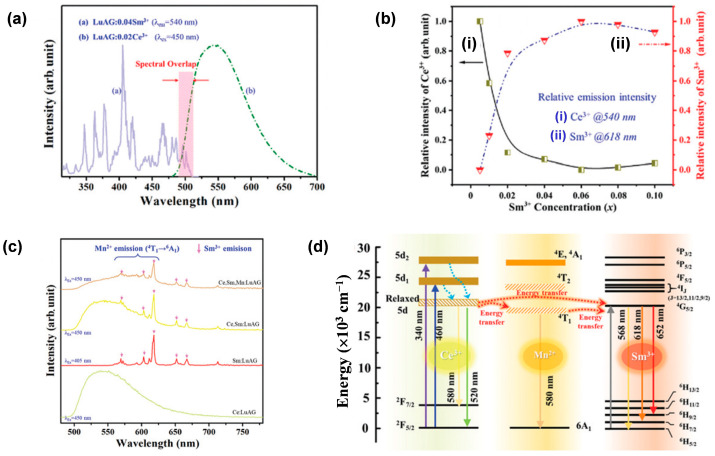
(**a**) PLE spectra (λ_em_ = 618 nm) of LuAG:0.04Sm^3+^ ceramics (blue line) and PL spectrum (λ_ex_ = 450 nm) of LuAG:0.02Ce^3+^ ceramics (red dashed line). (**b**) Relative intensity of Ce^3+^ (i) and Sm^3+^ (ii) of LuAG:0.02Ce^3+^,*x*Sm^3+^ as a function of Sm^3+^ concentration (*x*). (**c**) PL spectra of LuAG:0.02Ce^3+^, LuAG:0.04Sm^3+^, LuAG:0.02Ce^3+^,0.04Sm^3+^, and LuAG:0.02Ce^3+^,0.04Sm^3+^,0.04Mn^2+^ ceramics. (**d**) Schematic energy level diagram of Ce^3+^, Sm^3+^, and Mn^2+^. Reprinted with permission from ref. [77]. Copyright 2021, The Royal Society of Chemistry.

Due to the ^4^G_5/2_ → ^6^H*_J_* (*J* = 5/2 − 11/2) transitions of the 4f electrons of Sm^3+^ near-ultraviolet excitation, orange-red emission (>560 nm) may occur. However, Sm^3+^ is difficult to be excited by blue LD, limiting its applications in high-power wLD lighting. With this in mind, Sun et al. [77] doped Ce^3+^ and Sm^3+^ synchronously into LuAG to form highly transparent Lu_2.98−*x*_Ce_0.02_Sm*_x_*Al_5_O_12_ (LuAG:0.02Ce^3+^,*x*Sm^3+^) (*x* = 0.001–0.01) ceramics through vacuum sintering. The XRD pattern, the lattice parameter of LuAG:0.02Ce^3+^,*x*Sm^3+^ ceramics versus Sm^3+^ concentration, and the retrieved refinement of LuAG:0.02Ce^3+^,0.04Sm^3+^ ceramic have all evidenced the substitution of Ce^3+^ and Sm^3+^ for Lu^3+^ in the LuAG:Ce^3+^,Sm^3+^ ceramics. In addition, the spectral overlap between the PLE of LuAG:0.04Sm^3+^ and the PL of LuAG:0.02Ce^3+^ (Figure 14a), as well as the variation in emission intensity of Ce^3+^ (540 nm) and Sm^3+^ (618 nm) in (LuAG:0.02Ce^3+^,*x*Sm^3+^) with Sm^3+^ concentration (Figure 14b), confirmed the effective energy transfer from Ce^3+^ to Sm^3+^. To overcome the narrow bandwidth of Sm^3+^, Mn^2+^/Si^4+^ were further incorporated, taking advantage of the orange emission of Mn^2+^ and charge compensation of Si^4+^. The as-prepared single-structured Lu_3_Mn_0.04_Al_2.92_Si_0.04_O_12_:0.02Ce^3+^,0.04Sm^3+^ (LuAG:0.02Ce^3+^,0.04Sm^3+^,0.04Mn^2+^) ceramics displayed characteristic emission of Ce^3+^ (530 nm, 5d_1_ → 4f transition), Sm^3+^ (618 nm, ^4^G_5/2_ → ^6^H_7/2_ f-f transition), and Mn^2+^ (590 nm, ^4^T_1_ → ^6^A_1_ d-d transition), resulting in a broad emission spectrum spanning from 500 nm to 750 nm (Figure 14c). The spectral overlap between the PLE of LuAG:0.04Sm^3+^ and the PL of LuAG:0.04Mn^2+^, and the lifetime-decay studies of LuAG:0.02Ce^3+^,0.04Sm^3+^ and LuAG:0.02Ce^3+^,0.04Sm^3+^,0.04Mn^2+^ suggest multiple energy-transfer processes of Ce^3+^ → Mn^2+^, Ce^3+^ → Sm^3+^, and Mn^2+^ → Sm^3+^ (Figure 14d). When the LuAG:0.02Ce^3+^,0.04Sm^3+^,0.04Mn^2+^ ceramic was coupled with a 450 nm blue LD, the resulting wLD exhibited a CRI of up to 78.5.

## 5. Designing Composite Structural Ceramics

In addition to the above strategies, the design of composite structure ceramics, that is, the design of a double-layer converter with at least one layer of phosphor ceramic, can also broaden the emission spectrum, provided that the components of each layer and the structure between layers are subtly designed. In composite structural ceramics, the emission spectra range and color ratio of the two layers can be flexibly tuned, allowing improvement of the CRI. Moreover, they maintain the good thermal and mechanical performances of the parent ceramics. Therefore, the design of composite ceramics is a feasible way to achieve ceramics with high CRI values.

### 5.1. Combining Two Phosphor Ceramic Layers

**Figure 15:**
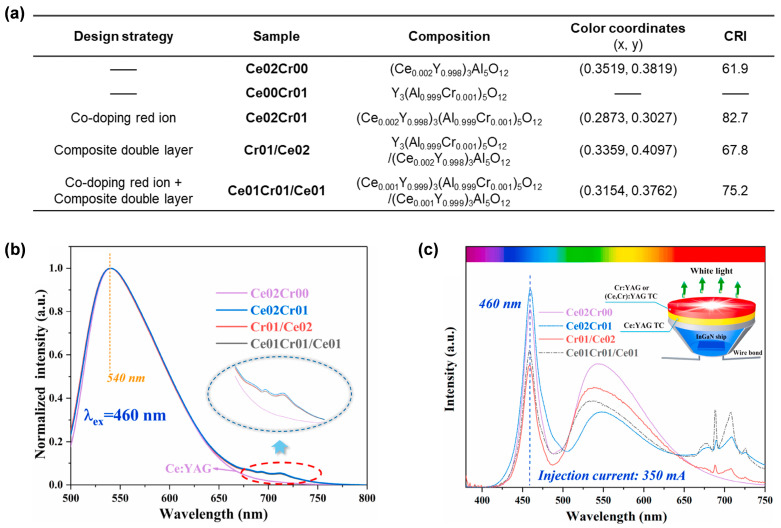
(**a**) Ingredients, color coordinates, and CRI of the prepared ceramics with different chemical components. (**b**) Normalized PL spectra of the corresponding ceramics under excitation at 460 nm. (**c**) Schematic diagram of composite structure ceramic-based wLED device (inset) and electroluminescence spectra of the corresponding ceramics. Reprinted with permission from ref. [78]. Copyright 2021, Elsevier.

To systematically investigate the influence of components and structure design on the properties of composite structural ceramics, Huang et al. [78] prepared double-layered YAG:Cr^3+^/YAG:Ce^3+^ (**Cr01/Ce02**) and YAG:Ce^3+^,Cr^3+^/YAG:Ce^3+^ (**Ce01Cr01/Ce01**) ceramics, as well as red-light-emitting Cr^3+^ co-doped YAG:Ce^3+^,Cr^3+^ (**Ce02Cr01**) single-layer ceramics (Figure 15a) through the vacuum sintering technique. As shown in Figure 15b, two characteristic emission bands centered at 540 nm and 687 nm appeared in the PL spectra for all ceramics containing Cr^3+^, which were attributed to the emission of Ce^3+^ and Cr^3+^, respectively. After the ceramics were packaged with 460 nm (2 W) blue LED chips, wLED devices were obtained and their electroluminescence spectra are shown in Figure 15c. In the yellow emission region (500–650 nm), the emission intensity of the single-layer **Ce02Cr01** ceramic is significantly lower than that of the composite double-layer **Cr01/Ce02** and **Ce01Cr01/Ce01** ceramics. The reduced LE of the **Ce02Cr01** ceramic can be ascribed to the energy consumption caused by the sensitization effect between Ce^3+^ ions and Cr^3+^ ions in this co-doping system. In the red emission region (650–750 nm), the emission intensity of **Cr01/Ce02** is lower than that of **Ce01Cr01/Ce01**, even though they contain the same Cr^3+^ content and have the same double-layer structure. The results show that the combined strategy of “co-doping” and “composite double layer structure” is more effective to improve the excitation efficiency of Cr^3+^, because the emitted light from the lower layer (**Ce01**) and the local layer (**Ce01Cr01**) of the Ce^3+^ ion can both be absorbed by the Cr^3+^ ion. This is further verified by the shorter lifetime of **Ce01Cr01/Ce01** (54.213 ns) than that of **Cr01/Ce02** (66.349 ns); in other words, the energy transfer efficiency of Ce^3+^ → Cr^3+^ increased in **Ce01Cr01/Ce01**. The CRI values of all the ceramics containing Cr^3+^ were elevated compared with YAG:Ce^3+^ (**Ce02Cr00**), and both **Ce02Cr01** and **Ce01Cr01/Ce01** ceramics exhibited CRI values beyond 75. The lower CRI of **Ce01Cr01/Ce01** (75.2) than **Ce02Cr01** (82.7) is due to mismatched red–green–blue ratio.

In another study reported by Lu et al. [60], the Al_2_O_3_-YAG eutectic was doped with Ce^3+^ and Cr^3+^ via a micro-pull-down method, respectively. When the Al_2_O_3_-YAG:Cr^3+^ was superimposed on the Al_2_O_3_-YAG:Ce^3+^ eutectic, the CRI of the Al_2_O_3_-YAG:Ce^3+^ and Al_2_O_3_-YAG:Cr^3+^ stacked LED reached 78.7.

Zhou et al. [79] also prepared LuAG:Ce^3+^,Cr^3+^ ceramics via solid-state sintering technique under vacuum condition. By combining YAG:Ce^3+^ ((Y_0.995_Ce_0.005_)_3_Al_5_O_12_) ceramic with LuAG:Ce^3+^,Cr^3+^ ((Lu_0.009_Ce_0.001_)_3_(Al_0.996_Cr_0.004_)_5_O_12_) ceramic, the ceramic-based wLED exhibited a CRI up to 88. Therefore, the “ceramic combination strategy” is highly promising for constructing high-quality wLEDs/wLDs.

### 5.2. Combining a Phosphor Ceramic Layer with a Free-Standing Phosphor Film

**Figure 16:**
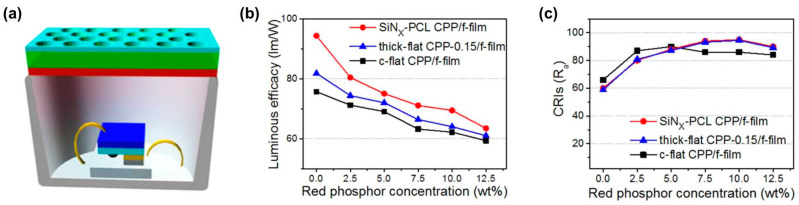
(**a**) Schematic diagrams of 2D PCL-assisted CPP/free-standing red film phosphor (SiN_X_-PCL CPP/f-film)-based LED. (**b**) LE (lm/W), and (**c**) CRIs of the LuAG:Ce^3+^ CPP/free-standing red film phosphor (c-flat CPP/f-film)-based LED, the SiN_X_-PCL CPP/f-film -based LED, and the thickness-increased CPP (0.15 mm)/free-standing red film phosphor (thick-flat CPP-0.15/f-film)-based LED as functions of the red phosphor concentration at equal current (350 mA). Reprinted with permission from ref. [80]. Copyright 2015, American Chemical Society.

Because the combination of a color conversion layer with green emission and a color conversion layer with red emission can produce high CRI, Park et al. [80] selected a green-emitting LuAG:Ce^3+^ ceramic phosphor plate (CPP) and a red-emissive (Sr,Ca)AlSiN_3_:Eu^2+^-silicone resin film to form composite structural ceramics. The red film was designed and prepared as a free-standing film (f-film) because it can make the phosphor less affected by the high temperature of the LED chip. In addition, a two-dimensional (2D) SiN_X_ photonic crystal layer (PCL) was introduced to improve the LE. All the fabricated wLEDs were color-converter-on-cup types, and different types of color converters, namely, the LuAG:Ce^3+^ CPP (c-flat CPP), the (Sr,Ca)AlSiN_3_:Eu^2+^ f-film, the 2D SiN_X_ PCL-assisted CPP (SiN_X_-PCL CPP), and the SiN_X_-PCL CPP/f-film (Figure 16a), were attached to the top of the LED cups with silicone resin. The comparative influence of the f-films with different concentrations of (Sr,Ca)AlSiN_3_:Eu^2+^ on the EL properties of LEDs capped with c-flat CPP, SiN_X_-PCL CPP, and thickness-increased CPP/f-film (thick-flat CPP-0.15/f-film) was investigated. The LE of SiN_X_-PCL CPP/f-film-based LEDs were always higher than those of the c-flat CPP/f-film and thick-flat CPP-0.15/f-film-based LEDs (Figure 16b), indicating that the 2D SiN_X_ PCL significantly increased the intensity of green emission. As shown in Figure 16c, the introduction of the f-films between the LuAG:Ce^3+^ CPP and the blue LED cup effectively improved the CRI. When the concentration of (Sr,Ca)AlSiN_3_:Eu^2+^ in the red film was over 7.5 wt%, the CRIs of both the SiN_X_-PCL CPP/f-film-based LED and the thick-flat CPP-0.15/f-film-based LED were over 90. Comprehensively, the SiN_X_-PCL CPP/f-film-based LED with a red phosphors concentration of 7.5 wt % exhibited an excellent CRI of 94 and an acceptable LE of 71.1 lm/W. Therefore, the combination of green-light-emitting phosphor ceramics, 2D PCL, and red phosphor f-film is effective to achieve high-power wLED with high CRI.

### 5.3. Coating a Phosphor/QDs Layer onto a Phosphor Ceramic Layer

**Figure 17:**
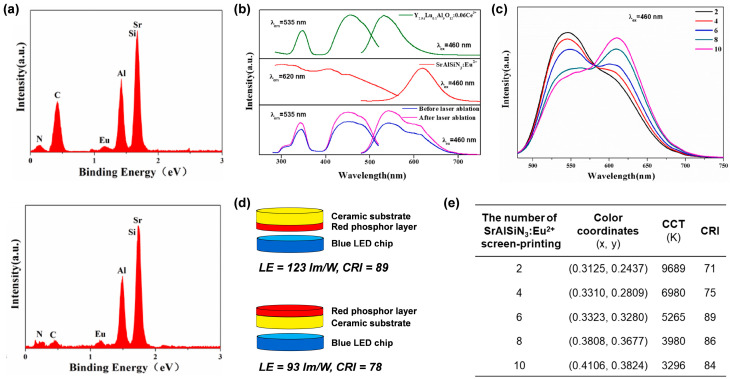
(**a**) EDS spectra of red phosphor layer before (**up**) and after (**below**) laser ablation. (**b**) PLE and PL spectra of Lu_0.1_Y_2.84_Al_5_O_12_:0.06Ce^3+^ ceramic, SrAlSiN_3_:Eu^2+^ phosphor, and composite ceramics before and after laser ablation. (**c**) PL spectra of the composite ceramics with different times of SrAlSiN_3_:Eu^2+^ screen-printing under 460 nm excitation. (**d**) Schematic drawing of the packaged W-LEDs with the red phosphor layer facing (**up**) and facing away from (**below**) the blue LED chip. (**e**) Color coordinates, CCT, and CRI of the wLED packaged with 1 W blue LED chip and composite ceramics with different numbers of SrAlSiN_3_:Eu^2+^ screen printings. Reprinted with permission from ref. [81]. Copyright 2022, Elsevier.

To obtain high-quality white lighting with a high CRI, Zhou et al. [81] proposed a method to coat red-emitting SrAlSiN_3_:Eu^2+^ slurry composed of SrAlSiN_3_:Eu^2+^ phosphor and transparent organic ink on green-emitting Lu_0.1_Y_2.84_Al_5_O_12_:0.06Ce^3+^ (LuYAG:Ce^3+^) ceramic substrate. Since SrAlSiN_3_:Eu^2+^ slurries contain organic matter which suffers poor thermal conductivity, they firstly coated the slurry on the LuYAG:Ce^3+^ ceramic using a screen-printing technique, and then removed the organic matter by laser ablation drying. According to the EDS spectra of the red phosphor layer (Figure 17a), the C element in the red phosphor was greatly reduced after laser ablation, confirming the removal of organic components by the laser ablation. Under 460 nm excitation, the composite ceramic exhibited a wide emission band of 500–675 nm (Figure 17b), which is due to the 5d_1_ → 4f transitions of Ce^3+^ and Eu^2+^ ions, respectively. It is worth noting that the emission intensity of the composite ceramic before laser ablation is much lower than that after laser ablation, which can be explained by the difference in refractive index between the organic matter and the SrAlSiN_3_:Eu^2+^ fluorescent powder. In addition, the emission spectrum can be modulated by adjusting the number of screen prints (Figure 17c). When the composite ceramics were combined with blue LED chips, it was found that the color coordinates, the CRI, and the CCT of the wLEDs were influenced by both the package pattern (Figure 17d) and the thickness of the red-emitting film (Figure 17e). When the red phosphor film was facing away from the blue LED chip, the yellow-green radiation emitted by the LuYAG:Ce^3+^ ceramic layer under excitation of the LED chip was absorbed by the red phosphor, resulting in decreases in LE and CRI. When the red phosphor layer was facing the 1 W blue LED chip, a wLED with CRI of 89 was obtained with a printing number of 6.

**Figure 18:**
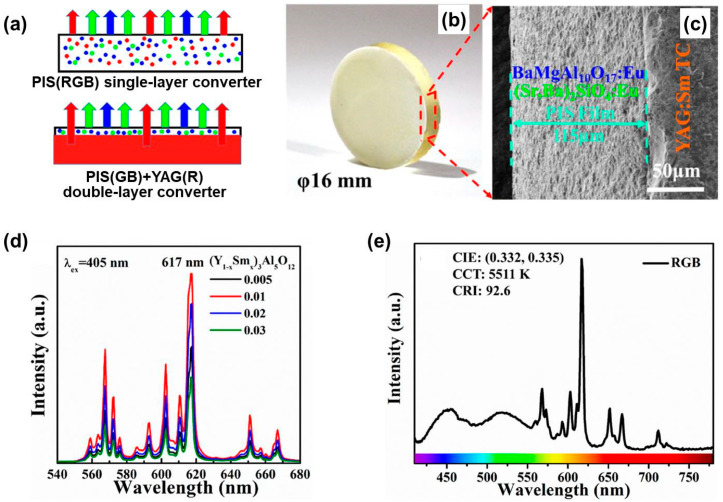
(**a**) Schematic diagrams of the single-layer converter (above) and double-layer converter (below). (**b**) Photograph and (**c**) cross-sectional SEM image of the double-layer white-light converter. (**d**) PL spectra of the (Sm*_x_*Y_1−*x*_)_3_Al_5_O_12_ (*x* = 0.005, 0.01, 0.02, and 0.03) ceramics under 405 nm excitation wavelength. (**e**) PL spectrum of the LED-driven tricolor converter under 405 nm excitation. Reprinted with permission from ref. [82]. Copyright 2019, American Chemical Society.

In another work described by Zheng et al. [82], a tricolor converter system combined with a 405 nm near ultraviolet chip was employed. In traditional tricolor converter systems, the nitride/fluoride red phosphors always show low thermal stability under high-power excitation and exhibit broad emission bands exceeding 650 nm that are difficult to perceived with human eyes, leading to a decrease in LE. To address the issue, the authors used vacuum sintering to prepare red-color-converter (Sm*_x_*Y_1−*x*_)_3_Al_5_O_12_ (*x* = 0.005, 0.01, 0.02, and 0.03) (YAG:Sm^3+^) ceramics with a narrow emission band and excellent thermal conductivity. A double-layer tricolor converter system was fabricated by coating a phosphor in silicone (PIS) film consisting of green-emitting (Sr,Ba)_2_SiO_4_:Eu^2+^ phosphor powders, blue-emitting BaMgAl_10_O_17_:Eu^2+^ phosphor powders, and organic silicon resin on the red-emitting YAG:Sm^3+^ ceramic. The blue/green-emitting PIS (PIS(GB))-coated YAG:Sm^3+^ samples were then hardened at 140 °C for 4 h to form the double-layer YAG(R)-PIS(GB) converter (Figure 18a,b). The cross-sectional SEM image (Figure 18c) shows that the PIS(GB) film is tightly bonded to the YAG:Sm^3+^ ceramic, effectively avoiding light-transmission loss and reducing interfacial thermal resistance. By modulating the concentration of Sm^3+^ in YAG:Sm^3+^ ceramics and fixing the thickness of the PIS(GB) film to 100 μm, the optical properties of the YAG(R)-PIS(GB) converter were optimized, and a CRI of 92.6 was achieved when *x* = 0.01 (Figure 18d,e). Compared with single-layer PIS(RGB) composed of red-emitting YAG:Sm^3+^, green-emitting (Sr,Ba)_2_SiO_4_:Eu^2+^ and blue-emitting BaMgAl_10_O_17_:Eu^2+^ phosphor powders, and organic silicon resin (Figure 18a), the YAG(R)-PIS(GB) converter exhibited higher heat transfer efficiency and avoided the blue emission from being self-absorbed by the red converter. Therefore, such a double-layer tricolor converter has broad application prospects in high-power lighting devices.

Xu et al. [83] coated a red-emitting quantum dot (QD) (λ_em_ = 634 nm) layer (CaSiAlN_3_:Eu^2+^) instead of red-emitting PIS on an Al_2_O_3_-YAG:Ce^3+^ ceramic plate to construct composite structural ceramics. The red-emitting QD was employed to overcome the luminescence saturation behavior of red-emitting phosphors at low power densities. Due to the excellent thermal conductivity of CPP, the coating of QD onto CPP can reduce the thermal erosion of the QD layer caused by laser irradiation. Under the irradiation of a blue laser (445 nm, 5 W), the surface temperature of the QD layer was as low as 68 °C, and no luminescence saturation occurred. When the CPP-QD was combined with a blue LD, the wLD device exhibited a CRI of up to 80, indicating the feasibility of applying CPP-QD in high-power wLD lighting.

## 6. Conclusions and Perspectives

In this review, we have provided a comprehensive overview of the preparation strategies for TPCs for the construction of high-power and high color rendering wLED/wLD lighting sources. These strategies can be primarily classified into four categories: host regulation, red-emitter doping, host regulation/red-emitter doping combination, and composite structure design. All the lighting systems exhibit high CRI (>75) with high stability and long lifespan. Additionally, their design principles, PL characteristics, and mechanisms for PL modulation were thoroughly discussed. This review addresses the previous lack of a summary of this research topic.

At present, the results of research in this area are still limited. On the one hand, we believe that the appropriate combination of the strategies summarized in this review can further improve device performance. On the other hand, although high-power and high-CRI wLED/wLD devices based on various TPCs have been successfully built, achieving the delicate balance between high chrominance and high LE remains a huge challenge. We believe that improving CRI while maintaining the high LE of wLED/wLD is a key goal for the next generation of high-power lighting. Moreover, in practical applications, in addition to LE and CRI, some other factors need to be considered. For instance, in multi-phase ceramics, taking Al_2_O_3_-YAG:Ce^3+^ as an example, it is usually difficult to achieve uniform distribution, which has a negative impact on the heat dissipation and uniformity of the luminescent centers of the ceramics. To address the issue, “unique” microstructures of these phosphor converters were developed to control light scattering and achieve color uniformity [84]. Homogeneous initial powder materials were obtained by regulating the excess of Al^3+^ of YAG:Ce^3+^ in the process of co-precipitation, which provided a possible way to optimize the composition uniformity of Al_2_O_3_-YAG:Ce^3+^ TPCs [85]. Many of these techniques to achieve high-quality TPCs are yet to be explored.

## Figures and Tables

**Table 1:** The ionic radii (Å) for a given CN of all relevant ions in this review refers to the YAG lattice.

Ions	CN = 4	CN = 6	CN = 8
Y^3+^	—	—	1.019
Al^3+^	0.39	0.535	—
Ce^3+^	—	1.01	1.143
Gd^3+^	—	0.938	1.053
Tb^3+^	—	0.923	1.04
Lu^3+^	—	0.861	0.977
Mg^2+^	0.57	0.72	0.89
Si^4+^	0.26	0.4	—
Cr^3+^	—	0.615	—
Mn^2+^	0.66	0.67	0.96
Pr^3+^	—	0.99	1.126
Sm^3+^	—	0.958	1.079
